# Changes of sarcopenia case finding by different Asian Working Group for Sarcopenia in community indwelling middle-aged and old people

**DOI:** 10.3389/fmed.2022.1041186

**Published:** 2022-11-07

**Authors:** Chun-Hung Ko, Hua-Ying Chuang, Shin-Jiuan Wu, Shou-Chun Yu, Yin-Fan Chang, Chin-Sung Chang, Chih-Hsing Wu

**Affiliations:** ^1^Department of Family Medicine, Chi Mei Medical Center, Tainan, Taiwan; ^2^Department of Food and Nutrition, Chung Hwa University of Medical Technology, Tainan, Taiwan; ^3^Department of Nursing, Chung Hwa University of Medical Technology, Tainan, Taiwan; ^4^Department of Internal Medicine, Chi Mei Medical Center, Tainan, Taiwan; ^5^Institute of Biomedical Sciences, National Sun Yat-sen University, Kaohsiung, Taiwan; ^6^Department of Medical Research, Chi Mei Medical Center, Tainan, Taiwan; ^7^Department of Family Medicine, National Cheng Kung University Hospital, College of Medicine, National Cheng Kung University, Tainan, Taiwan; ^8^Department of Family Medicine, College of Medicine, National Cheng Kung University, Tainan, Taiwan; ^9^Institute of Gerontology, College of Medicine, National Cheng Kung University, Tainan, Taiwan

**Keywords:** Asian Working Group for Sarcopenia, sarcopenia, gait speed, hand grip, skeletal muscle index, calf circumference

## Abstract

Sarcopenia is an emerging issue, but there is no universal consensus regarding its screening and diagnosis, especially regarding the influence of the Asian Working Group for Sarcopenia (AWGS) 2019 new definition on the prevalence of community-dwelling adults. To compare the prevalence of sarcopenia between the 2019 and 2014 definitions, a cross-sectional study including 606 normal nutritional status subjects (203 men/403 women; mean age 63.3 ± 10.0 years) was performed. Sarcopenic parameters, including calf circumference, grip strength, 6-m gait speed, and bioelectrical-impedance-analysis-derived skeletal mass index (SMI), were evaluated. According to the 2019 AWGS definition, the prevalence of possible sarcopenia and sarcopenia among community-dwelling adults was 7.4 and 2.8%, respectively. There were highly consistent findings regarding sarcopenia between the 2019 and 2014 AWGS definitions according to Cohen's kappa coefficient (0.668). However, the prevalence of possible sarcopenia according to 2014 and 2019 AWGS in males increased 7.9%; in contrast, sarcopenia decreased from 7.4 to 3.7% in females (*p* < 0.001). In conclusion, the AWGS 2019 definition is more convenient for sarcopenia case screening and remains considerably consistent in sarcopenia identification in community-dwelling adults in Taiwan. The discordance of possible sarcopenia and sarcopenia by sex is a concern.

## Introduction

Sarcopenia is a progressive age-related decline of skeletal muscle (1). As the rapid growth of the elderly population, sarcopenia has become the most important issue in public health (2). As the COVID-19 outbreak began in 2020, all people were forced to stay at home and decrease their daily activity, and some gym and leisure activities were stopped (3–5). Reduced activity and subsequent weight gain cause chronic diseases (6), including frailty and sarcopenia, to worsen due to COVID-19 isolation/quarantine or hospitalization, particularly in elderly individuals (7). As much as 8% muscle strength decrease after short-term reduced physical activity for 14 days in the elderly with low muscle mass (8). The restricted quarantine also worsen the adverse consequences of sarcopenia such as functional decline, physical disability, falls, impaired quality of life, increased health care expenditures, hospitalization and death (9–12). Therefore, the finding and intervention of the possible sarcopenia as early as possible is an emerging concern during and after the COVID-19 pandemics (13, 14). The Asian Working Group for Sarcopenia (AWGS) (15, 16) recommended more attention and active intervention to enhance resilience, especially during the increase in sarcopenia owing to the aging society and COVID-19 pandemic.

The prevalence of sarcopenia varies due to differences in lifestyle habits, environment, culture, and ethnicities (17–19). In community-based studies, the prevalence of sarcopenia was 12.5% in Belgium, 7.5% in Japan, 4.8% in Brazil and 4.5% in Germany (20–24). The prevalence of sarcopenia ranges from 3.9% (2.5% in women and 5.4% in men) to 7.3% (6.5% in women and 8.2% in men) among community-dwelling elderly adults in Taiwan (25). Worldwide, the prevalence of sarcopenia in community-dwelling elderly adults is ~10.0% (26). The different operational definition is one of the major reasons of diverse prevalence (26).

Based on AWGS 2014, a new AWGS algorithm was declared in 2019. The simple, non-invasive, and inexpensive measurement of calf circumference (CC) was recommended as a first-line screening tool. Gait speed ranged from 0.8 to 1.0 m/s, and handgrip strength in males ranged from 26 to 28 kg (27).

The variation between the EWGSOP and EWGSOP2 criteria has also been reported in many studies, as a lower number of males and a lower prevalence of the combination of low muscle mass and muscle strength were found when EWGSOP2 was used (28, 29). On the other hand, a comparison between the AWGS 2019 and AWGS 2014 criteria revealed more older males who required long-term care, and the prevalence of sarcopenia was 16% in prefrail community-dwelling older adults based on the AWGS 2019 (30, 31). Even though these is no consistent conclusion about the comparison between EWGSOP1 and EWGSOP2 (32–35), most of the experts endorsed new EWGSOP2 as well (32, 36, 37). In contrast to EWGSOP series, limited studies explored the clinical utility regarding both the 2014 and new 2019 Asian Working Group for Sarcopenia. We hypothesized that the new AWGS 2019 criteria might impact sarcopenia identification. This study scrutinized the differences in the sarcopenia ratio between the AWGS 2019 and 2014 criteria for community-dwelling adults.

## Participants and methods

Using the convenient sampling method, we surveyed 606 community-dwelling middle-aged and elderly Taiwanese individuals who were ambulatory and lived in Jia-Li District, southern Taiwan from May 2016 to June 2017. The exclusion criteria were uncontrolled hypertension or diabetes, stroke, severe liver or renal disease, gastrointestinal disease, neuromuscular disease, infectious disease, pulmonary disease, endocrine system disease, neurological or acute/advanced psychiatric disease, cancer, a history of seizures, and sensitivity to any study procedures. At the initial screening, recruits underwent a review of the inclusion and exclusion criteria, the validated structural questionnaires, which included basic characteristics, smoking and drinking habits, medical history, concomitant therapies, daily activity evaluation, and anthropometric and body composition measurements (38–40). We defined participants to have a smoking habit if they had smoked more than 100 cigarettes and still smoked one pack (20 cigarettes) at least per month for more than 6 months, and alcohol drinking was defined by if participants still drank one time per week for more than 6 months (39, 41). A history of hypertension, dyslipidemia or diabetes was assessed by referring to the self-reported physician's diagnosis. The short form Mini-Nutritional Assessment (MNA) was used to evaluate nutritional status (42, 43).

Bodyweight and standing height were measured using a medical weight- and height-analyzing scale (Detecto™, Webb City, MO, USA); participants were barefoot and dressed in light clothing. Body mass index (BMI) was calculated using the following formula: BMI = bodyweight in kg/height in m^2^. CC was measured at the greatest girth region of both calves while participants were in a seated and relaxed position with knee flexion at 90° using inelastic tape (44). Functional limitations were assessed by using the Short Physical Performance Battery (SPPB) (45). A higher summary performance score represents better performance and vice versa (46). A single-frequency 8-electrode bioelectrical impedance analysis (BIA) device (BC-418; Tanita Corp., Itabashi-ku, Tokyo, Japan) was used to measure body composition, including body fat and skeletal muscle mass (SMM) (47) [estimated using Janssen's equation (SMI = kg/m^2^)] (1, 41, 48). Gait speed was measured by the walking test modified to a 6-m distance (49). Grip strength [Grip-D (TKK 5401); Japan] was assessed in two separate 30-s fast-twist tests for one hand for each participant. The maximum value of grip strength was used to indicate muscle function (50). The definition of a pre-state of sarcopenia termed “presarcopenia” is low muscle mass with normal physical function and performance by EWGSOP. These varieties were compared between AWGS 2019 and AWGS 2014 (27, 51).

### Ethical approval

The study (ClinicalTrials.gov Identifier: NCT03891134) was approved and monitored by the Institutional Review Board of the Chi Mei Medical Center (CMMC10504-J01). Each participant was informed of the purpose of the study, experimental procedures, and potential risks after providing signed written consent.

### Statistics

All statistical analyses were performed using the Statistical Package for the Social Sciences 22 for Windows (IBM Corp., Armonk, NY, USA). Categorical and continuous variables are expressed as numbers (percentages) and as the means ± standard deviations (SDs), as indicated. Continuous variables were analyzed using unpaired t tests, and categorical variables were analyzed using the χ^2^ test. Significance was set at *p* < 0.05 for two-tailed analysis. The clinical characteristics and measurement data of males and females were compared by unpaired t tests. Regarding the comparison of the number of cases of sarcopenia and those with handgrip strength and usual gait speed measurements below the reference values between AWGS 2019 and AWGS 2014, we performed McNemar tests to compare sexes. To distinguish the corresponding reliability between the AWGS 2019 and AWGS 2014, Cohen's kappa coefficient (κ) was used to assess interrater agreement for categorical data (52). The sensitivity and specificity were calculated accordingly.

## Results

**Figure 1:**
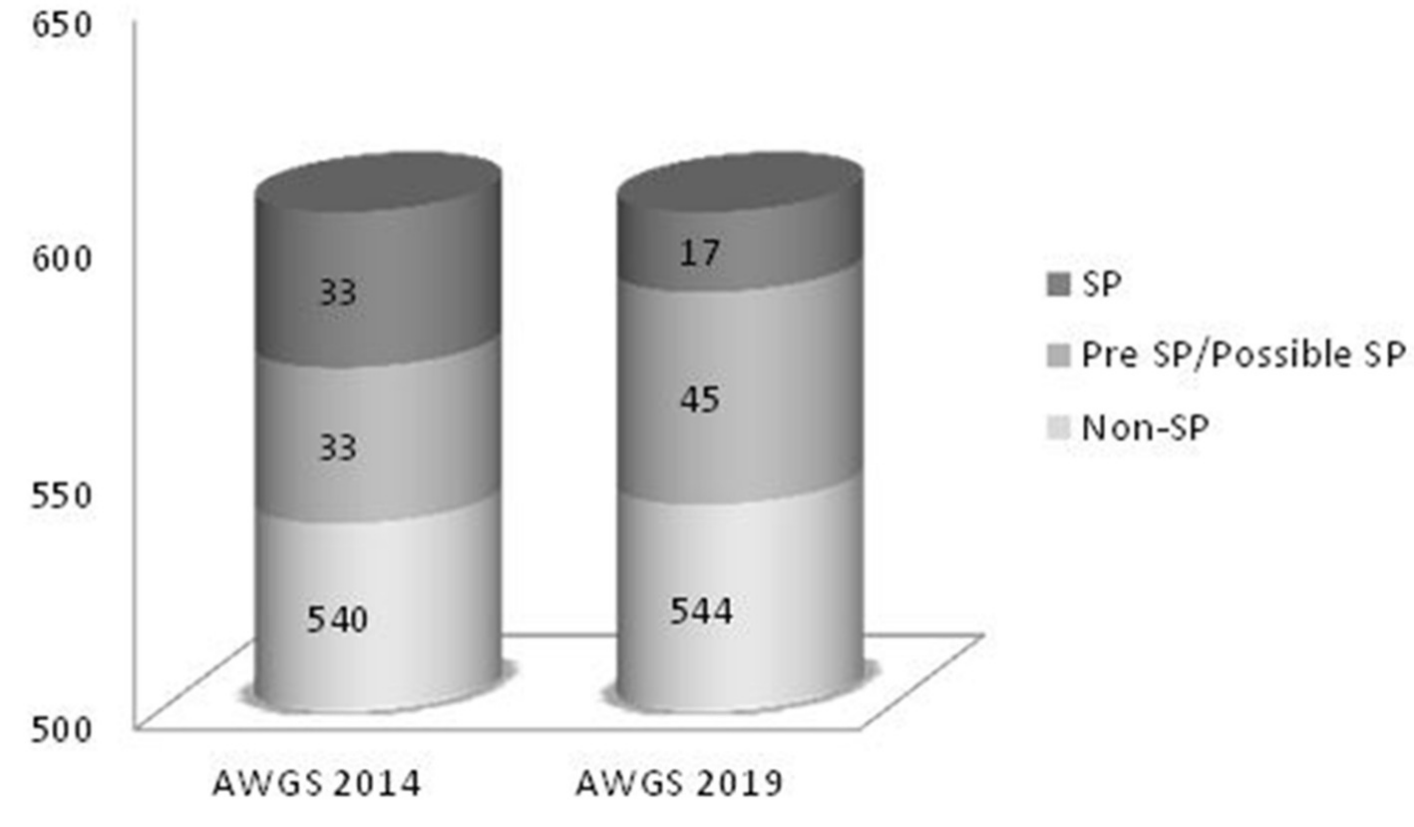
Conceptual distribution of sarcopenia. SP, sarcopenia; Possible Sarcopenia on 2019 AWGS, Pre-Sarcopenia on 2014 AWGS.

A total of 606 candidates (203 males, 403 females) with a mean age of 63.3 ± 10.0 years were identified. The distribution of sarcopenia in 2014 and 2019 is presented in Figure 1. There were 3.1% (AWGS 2014) and 2.5% (AWGS 2019) severe sarcopenia cases.

Table 1 shows the demographic characteristics and measurements of the study participants according to the definition of 2019 AWGS. The mean MNA and SPPB scores indicated the participants with normal nutritional status and without physical function limitation. Table 2 outlines the ratios of overall participants and participants grouped by sex according to the AWGS 2014 and 2019 definitions. The mean prevalence of possible sarcopenia and sarcopenia was 7.4 and 2.8% according to the 2019 criteria, compared to, 5.4 and 5.4% using the 2014 definition, respectively. In males, on average, 7.9% of participants had possible sarcopenia based on the 2019 criteria, compared to 0% using the 2014 definition (presarcopenia). In females, on average, 7.2 and 3.7% of participants had possible sarcopenia and sarcopenia based on the 2019 criteria, compared to 8.2 and 7.4% using the 2014 definition. There was slight agreement on nonsarcopenia and possible sarcopenia (κ: 0.179) but substantial agreement on sarcopenia (κ: 0.668; Table 3). Compared to the 2014 criteria, the 2019 AWGS criteria sensitivity and specificity were 51.5 and 100%, respectively (Supplementary Table 1).

**Table 1:** Demographic characteristics of 606 Taiwanese adults at baseline.

	**Total**	**Male**	**Female**	***p*-Value**
Case no	606	203	403	
Age, mean ± SD (years)	63.3 ± 10.0	64.8 ± 9.4	62.5 ± 10.2	0.005
Height, mean ± SD (cm)	158.6 ± 7.8	165.5 ± 6.7	155.1 ± 5.8	<0.001
Weight, mean ± SD (kg)	61.0 ± 11.1	66.9 ± 10.7	58.1 ± 10.1	<0.001
Body mass index, mean ± SD (kg/m^2^)	24.2 ± 3.7	24.4 ± 3.3	24.1 ± 3.9	0.358
Calf circumference, mean ± SD (cm)	35.1 ± 3.9	35.8 ± 3.1	34.8 ± 4.2	0.001
Skeletal muscle index, mean ± SD (kg/m^2^)	7.18 ± 1.40	8.71 ± 0.88	6.40 ± 0.88	<0.001
Habitual smoking (%)	13.0	32.0	3.4	<0.001
Alcohol drinking (%)	10.8	27.2	2.4	<0.001
Education (%)^†^	57.8	70.5	51.4	<0.001
History of hypertension (%)	31.7	34.8	30.1	0.341
History of diabetes (%)	15.0	15.0	14.9	0.847
History of dyslipidemia (%)	8.6	10.5	7.6	0.397
Mini-Nutrition Assessment score, mean ± SD	13.1 ± 2.0	13.0 ± 2.0	13.1 ± 1.9	0.338
Gait speed, mean ± SD (m/s)	0.90 ± 0.26	0.92 ± 0.24	0.89 ± 0.27	0.108
Handgrip, mean ± SD (kg)	25.5 ± 8.7	34.1 ± 8.6	21.2 ± 4.6	<0.001
SPPB score, mean ± SD	10.3 ± 1.8	10.5 ± 1.6	10.2 ± 2.0	0.095

Comparison between male and female, Continuous variables: unpaired T-test, Categorical variables: χ^2^-test, statistically significant: p-values < 0.05.

SPPB, short physical performance battery; SD, standard deviation.

† Beyond elementary school.

**Table 2:** Prevalence and difference of non-sarcopenia, pre/possible sarcopenia, and sarcopenia in Taiwanese community-dwelling adults using the cut-of values from the 2014 and 2019 Asian Working Group for Sarcopenia (AWGS) definition, stratified by sex.

**Sex, *n* (%)**	**AWGS 2019**	**AWGS 2014**	**Difference^#^**	***p*-Value**
**Overall (*****n*** **= 606)**				
Non-sarcopenia	544 (89.8%)	540 (89.1%)	0.7%	0.757
Possible/Presarcopenia	45 (7.4%)	33 (5.4%)	2.0%	0.213
**Sarcopenia**	17 (2.8%)	33 (5.4%)	0%	<0.001
**Males (*****n*** **= 203)**				
Non-sarcopenia	185 (91.1%)	200 (98.5%)	−7.4%	<0.001
Possible/Presarcopenia	16 (7.9%)	0 (0%)	7.9%	–
**Sarcopenia**	2 (1.0%)	3 (1.5%)	−0.5%	1.000
**Females (*****n*** **= 403)**				
Non-sarcopenia	359 (89.1%)	340 (84.4%)	4.7%	0.040
Possible/Presarcopenia	29 (7.2%)	33 (8.2%)	−1.0%	0.704
**Sarcopenia**	15 (3.7%)	30 (7.4%)	−3.7%	<0.001

McNemar test for AWGS 2019 criteria versus AWGS 2014 criteria, statistically significant: p-values < 0.05.

# Difference indicates the difference in prevalence calculated using the AWGS 2014 subtracted by that calculated using the AWGS 2019.

**Table 3:** Consistency between 2019 and 2014 AWGS definition in Taiwanese community-dwelling adults.

**Definition (*****n***, ***κ*****)**		**2019**		
		**Non-sarcopenia**	**Possible sarcopenia**	**Sarcopenia**
2014	Non-sarcopenia	495 (0.179)^*^	45 (−0.042)^*^	0 (−0.058)^*^
	Pre-sarcopenia	49 (−0.042)^*^	17 (0.179)^*^	17 (0.382)^*^
	Sarcopenia	16 (−0.053)^*^	17 (0.308)^*^	17 (0.668)^*^

The correlation of 2019 and 2014 AWGS criteria by kappa consistence test, κ: Kappa coefficient.

* Statistically significant, p-values < 0.001.

AWGS, Asian Working Group for Sarcopenia.

## Discussion

When the AWGS 2019 algorithm was announced, the prevalence of sarcopenia varied since the handgrip strength of males and gait speed increased. When we used the 2019 AWGS definition, the prevalence of sarcopenia was 2.8% within the range of community settings (1–30%) (16, 27) but lower than that when the 2014 definition was used (5.4%). The decrease in the prevalence of sarcopenia cases was predominantly observed among females (3.7%). Sex-based differences of a lower prevalence in females and a higher prevalence in males were found for possible sarcopenia according to the AWGS 2019 definition. There was higher relative consistency in sarcopenia between the 2019 and 2014 AWGS definitions in South Taiwanese community-dwelling adults.

The prevalence of pre-sarcopenia according to AWGS was 8.4% in Chinese T2DM elderly (53). However, some studies reported the prevalence of possible sarcopenia based on the 2019 AWGS criteria widely ranged from 2.9 to 38.5% in Asian (54–56). During the COVID-19 pandemic, one study demonstrated the skeletal muscle mass decrease by life space assessment (57). The pooled prevalence of sarcopenia in COVID-19 was 48.0% in one meta-analysis study (58). As the subjects at risk of sarcopenia would increase rapidly in post COVID-19 pandemic era, it is plausible to ascertain the utility of case finding by 2019 AWGS.

In the present study, we found no significant statistical difference in the prevalence of non-sarcopenia and pre-sarcopenia/possible sarcopenia between 2014 and 2019 AWGS algorithm, but higher possible sarcopenia as revealed in previous study with EWGSOP2 (59). The 2019 AWGS algorithm initiated the case finding from calf circumference, SAR-F or SARF-CALF coordinated with muscle strength instead of muscle mass in 2014 AWGS for initial identifying subjects at risk of sarcopenia. That is, the new 2019 algorithm reached the purpose of case finding in community-dwelling middle aged and old adults at risk of sarcopenia in a more user-friendly manner. The AWGS 2019 algorithm recommended either calf circumference or the SARC-F or the SARC-Calf questionnaires as an initial screening tool for case finding in the community or primary health care (27). When the subjects' CC was below the standard values (male: 34 cm, female: 33 cm), further assessment and diagnostic procedures were arranged. Screening for sarcopenia using CC is an accurate and easy (60) and has moderate-to-high sensitivity and specificity (61–65). We found that CC alone as initial case finding may result in underestimation in women of possible sarcopenia. In one trial of cadavers (66), CC showed a higher correlation with muscle mass in males but not in females because of the higher fat mass among females (67). CC adjusted for BMI was proposed to predict sarcopenia (68). Lower sensitivity and higher specificity were calculated in our assessment, and the result was similar to that of J Reiss et al. about EWGSOP2 (28) and consistent with a lower Kappa coefficient in possible/presarcopenia. Some studies found low sensitivity but high specificity for SARC-F (69, 70); however, SARC-Calf (SARC-F combined with CC) with improved sensitivity (71) would be a clinical practice tool for identifying sarcopenia but would require more time completing questionnaires. Ethnic differences in sarcopenia and the difference between EWGSOP1 and EWGSOP2 have been reported (28, 29), and more trials analyzing AWGS 2019 will be helpful for sarcopenia early detection and intervention (30, 31).

Our study had limitations. First, this cross-sectional study was initiated before the announcement of the EWGSOP2 and 2019 AWGS. Additionally, the SARC-F and SARC-Calf were not recommended for identifying sarcopenia. The lack of consistent analysis could occur in the absence of SARC-F and SARC-Calf information. In our study, although the non-sarcopenia and possible sarcopenia rates were similar between the 2014 and 2019 definitions, sex differences and female predominance in our study might affect the prevalence and limit generalizability to other trials (72). Second, the enrolled adults were engaged in agricultural work in rural areas of south Taiwan. There might be a difference compared to adults living in the city. We believe the most valuable finding is that when subjects are judged as having sarcopenia according to the 2019 definition, the possibility of severe sarcopenia may be higher. More comparisons are needed to confirm our findings and investigate the bias.

In conclusion, this study revealed that the AWGS 2019 algorithm was convenient and efficient in sarcopenia case identification for community-dwelling adults in Taiwan. However, sex differences in possible sarcopenia/sarcopenia should not be ignored when conducting comparisons with the 2014 criteria, and the sarcopenia rate remained considerably consistent.

## Data availability statement

The original contributions presented in the study are included in the article/Supplementary material, further inquiries can be directed to the correspondingauthor.

## Ethics statement

The studies involving human participants were reviewed and approved by Chi Mei Medical Center (CMMC10504-J01). The patients/participants provided their written informed consent to participate in this study.

## Author contributions

Conception and design of the study: C-HK, H-YC, C-SC, and C-HW. Statistical analyses: C-HK, S-CY, and C-SC. Acquisition of data: S-JW, Y-FC, and C-SC. Drafting the article: C-HK and C-HW. Research data interpretation, suggestions and discussion, critical revision of the manuscript, and final approval of the manuscript: all authors.

## Funding

We are grateful to Taiwanese Osteoporosis Association, the Taiwan Ministry of Science and Technology (MOST No. 106-2314-B-006-064-MY2), Chi Mei Hospital (Holistic Health Care No. JCHCR 10510), and National Cheng Kung University Hospital (No. NCKUH-10709012) for the listed grants.

## Conflict of interest

The authors declare that the research was conducted in the absence of any commercial or financial relationships that could be construed as a potential conflict of interest.

## Publisher's note

All claims expressed in this article are solely those of the authors and do not necessarily represent those of their affiliated organizations, or those of the publisher, the editors and the reviewers. Any product that may be evaluated in this article, or claim that may be made by its manufacturer, is not guaranteed or endorsed by the publisher.
