# Supplementary material for: Changes of sarcopenia case finding by different Asian Working Group for Sarcopenia in community indwelling middle-aged and old people
Source: Front Med (Lausanne). 2022 Nov 7;9:1041186. doi: 10.3389/fmed.2022.1041186 (PMC9680091; doi:10.3389/fmed.2022.1041186)
Supplement: Supplementary file 1 [file Table_1.pdf]

Supplement Table 1: The sensitivity and specificity of sarcopenia and non-sarcopenia between 2014 and 2019 AWGS definition.

No		2014		
		Sarcopenia	Non-sarcopenia	Total
2019	Sarcopenia	17	0	17
	Non-sarcopenia	16	495	511
	Total	33	495	528

AWGS: Asian Working Group for Sarcopenia

Sensitivity=17/33=0.515

Specificity=495/495= 1
